# Inhibitor of Apoptosis Protein-Like Protein-2 as a Novel Serological Biomarker for Breast Cancer

**DOI:** 10.3390/ijms131216737

**Published:** 2012-12-07

**Authors:** Mingjun Xiang, Wei Zhou, Dandan Gao, Xiansong Fang, Qian Liu

**Affiliations:** 1 Department of Biochemistry & Molecular Biology, College of Medical Science, Jishou University, Jishou 416000, China; 2 Department of Biochemistry & Molecular Biology, Gannan Medical University, Ganzhou 341000, China; 3 Hunan Provincial Key Laboratory for Biology and Control of Plant Diseases and Insect Pests, College of Bio-Safety Science & Technology, Hunan Agricultural University, Changsha 410128, China; E-Mail: mengrzhou@163.com; 4 Institute of Medical Biology, Chinese Academy of Medical Sciences, Kunming 650118, China; E-Mail: ddgao2008@imbcams.com.cn; 5 Clinical Laboratory, the First Affiliated Hospital, Gannan Medical University, Ganzhou 341000, China; E-Mail: changmengjin@sina.com

**Keywords:** ILP-2, breast cancer, serological biomarker

## Abstract

Inhibitor of apoptosis protein-like protein-2 (ILP-2) has only been detected in the testis and in lymphoblastoid cells. Although previous studies have not reported the presence of ILP-2 in breast cancer tissues, this study indicates the presence of ILP-2 in breast cancer serum samples. To validate whether ILP-2 is a novel serological biomarker for breast cancer, we conducted two-dimensional gel electrophoresis (2DE) and matrix-assisted laser desorption/ionization-time of flight mass spectrometry analysis on 400 breast cancer serum samples and 40 non-cancer serum samples (*i.e.*, healthy controls). We then performed a Western blot analysis of 10 breast cancer serum samples and 10 non-cancer serum samples. Finally, we analyzed 35 serum samples from healthy controls or subjects with breast cancer, other types of cancer, galactophore hyperplasia or breast cancer post-surgery by using 2DE and enzyme-linked immunosorbent assay. Our results indicate that ILP-2 is a novel breast cancer biomarker in the peripheral blood.

## 1. Introduction

Breast cancer is the most common type of cancer that affects women and accounts for 23% of all cancers [1]. The incidence of breast cancer is estimated to increase by approximately 0.5% annually [1]. Despite improvements in breast cancer therapy, more than one-fourth of patients diagnosed with breast carcinoma eventually die from the disease [2]. Thus, early diagnosis and effective therapies for breast cancer are necessary.

The use of biomarkers is an effective diagnostic method for breast cancer. However, a limited number of biomarkers for breast cancer have been validated for clinical application [3]. Therefore, the identification of a novel biomarker related to breast oncogenesis is essential.

Inhibitors of apoptosis proteins (IAPs) are apoptosis inhibitors that may protect against apoptotic stimuli and suppress apoptotic cell death [4]. Eight human IAP proteins have been identified: XIAP [5,6], c-IAP1 [7], c-IAP2 [7], NAIP [8], ML-IAP [9,10], survivin [11,12], BRUCE [13] and inhibitor of apoptosis protein-like protein-2 (ILP-2) [14,15]. ILP-2 is the most recent member of the human IAP family to be identified [14,15].

ILP-2 has been mapped to chromosome 19q13.3–13.4 [14]. This biomarker has only been detected in humans and great apes [14]. In normal tissues, ILP-2 gene expression has only been detected in the testis and in lymphoblastoid cells [14]. ILP-2 over-expression did not protect against Fas- or tumor necrosis factor-mediated cell death, but prevented cell death induced by Bax or caspase-9 *in vitro*[14,15].

Although no study has reported ILP-2 expression in breast cancer tissues, our results indicate the presence of ILP-2 in breast cancer serum samples. Questions, such as why ILP-2 exists in breast cancer serum samples, is there a correlation between ILP-2 and breast carcinogenesis and is ILP-2 is novel serological biomarker for breast cancer, remain unanswered.

To validate the correlation between ILP-2 and breast cancer and to determine whether ILP-2 is a novel biomarker for breast cancer, we first applied comparative proteomics and immunological methods to breast cancer and non-cancer serum samples. Subsequently, we performed the same procedures on serum samples from subjects with other types of cancer, galactophore hyperplasia and breast cancer post-surgery. Our results suggest that ILP-2 is a novel serological biomarker for breast carcinogenesis.

## 2. Results and Discussion

### 2.1. Comparison of Serum Proteins with 2DE

**Figure 1:**
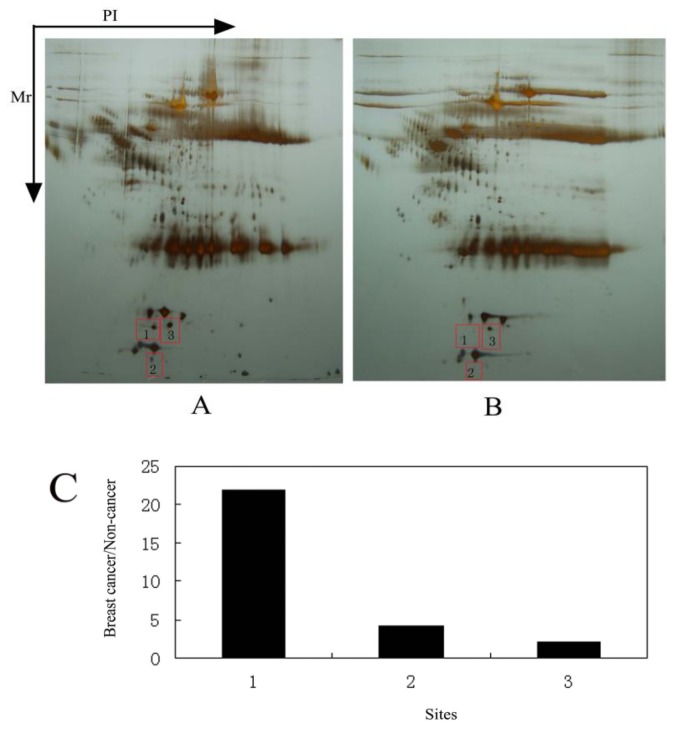
2D PAGE results of the serum samples from breast cancer patients and healthy females. (**A**) 2D PAGE protein spot map from the breast cancer patients; (**B**) 2D PAGE protein spot map of the healthy females; (**C**) Ratio of serum protein levels in the breast cancer serum sample to the non-cancer control sample.

The serum proteomic analysis was performed with 2DE. Two iterations showed the reliability and reproducibility of the experimental results. More than 100 protein spots were thoroughly separated, although highly abundant proteins interfered with the map (Figure 1A,B). Comparing the 2DE map of the breast cancer serum sample with that of the non-cancer serum sample, we observed three protein spots in the breast cancer serum sample that showed a two-fold or greater change in expression level (Figure 1A,B). Protein spots 1, 2, and 3 were increased in the breast cancer sample by 21.9-, 4.2- and 2.03-fold, respectively (Figure 1C).

### 2.2. Peptide Mass Fingerprinting Analysis

**Figure 2:**
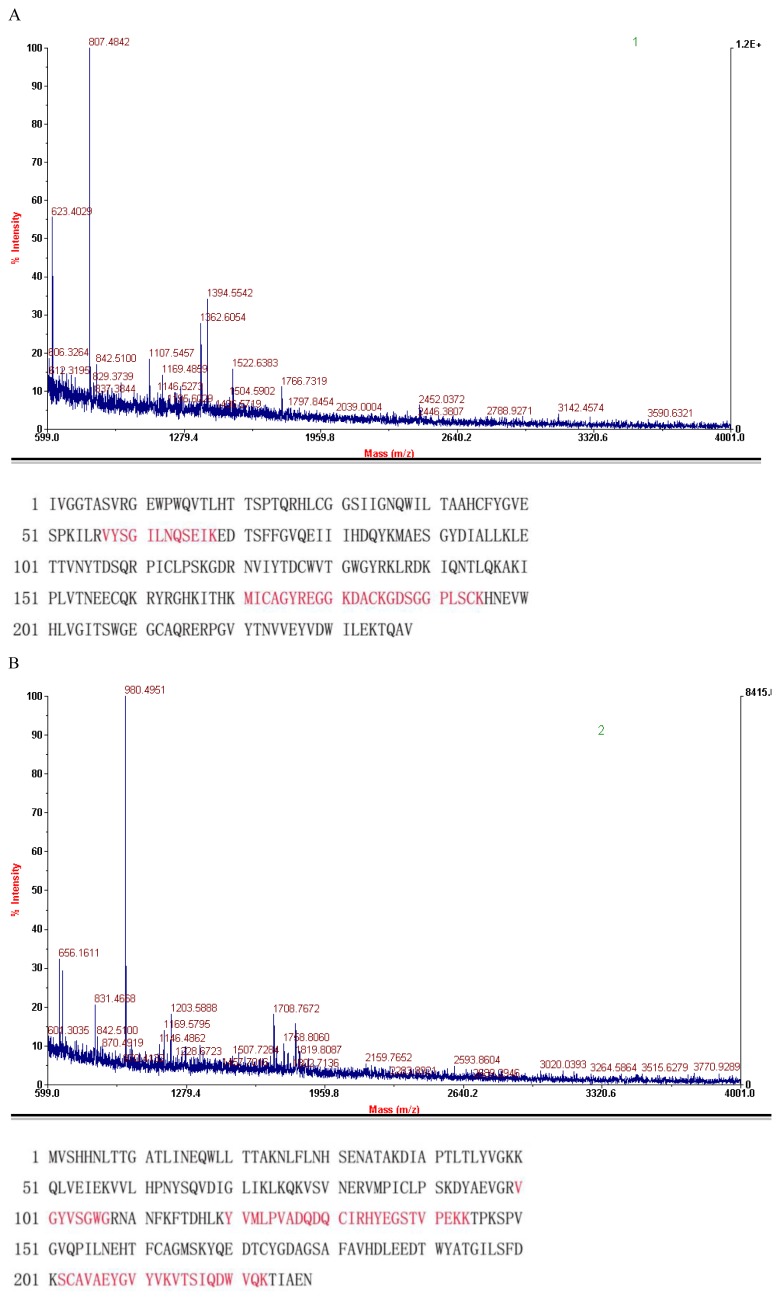

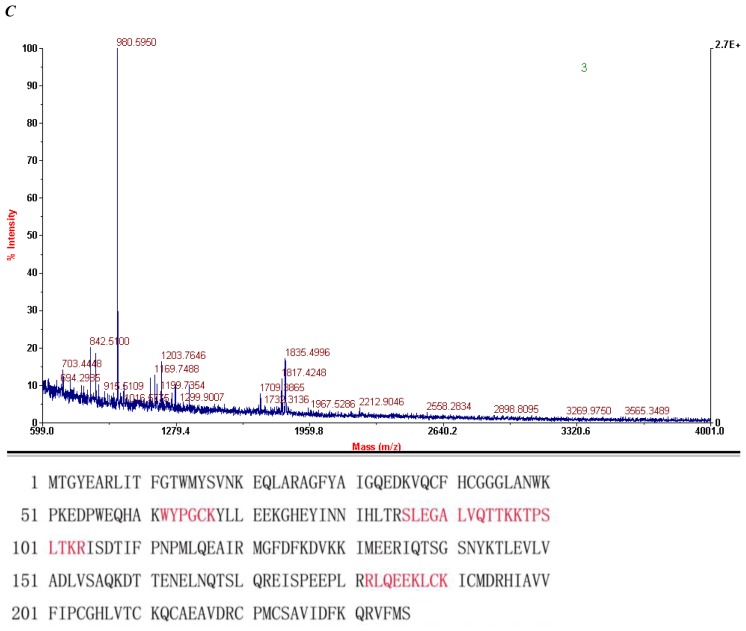
Mass spectra results of the identified proteins. The red peptides denote the sequence that was confirmed by mass spectrometry analysis. (**A**) The Fxia catalytic domain; (**B**) HP protein; (**C**) ILP-2.

The three protein spots that were significantly increased in the breast cancer serum sample gel were excised and analyzed with MALDI-TOF-MS to obtain the peptide sequences (Figure 2).

Using the NCBI GenBank database, we matched the peptides to three protein sequences (Figure 2). The proteins were identified as the human crystal structure of the Fxia catalytic domain in complex with ecotinm84r (spot 1 in Figures 1 and 2A), the haptoglobin alpha (2FS)-beta precursor (HP) (spot 2 in Figures 1 and 2B) and ILP-2 (spot 3 in Figures 1 and 2C) (see Table 1 for details).

### 2.3. Western Blot Analysis of the ILP-2 Levels in the Breast Cancer and Non-Cancer Serum Samples

**Figure 3:**
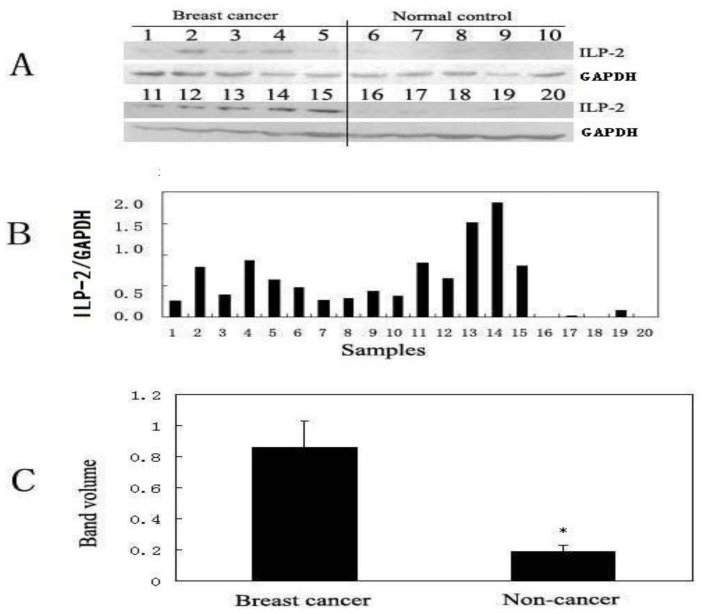
Western blot analysis of the individual serum samples from breast cancer patients and control subjects. (**A**) ILP-2 Western blots of the breast cancer serum samples and the non-cancer serum samples; (**B**) ILP-2 expression levels normalized to GAPDH; (**C**) Expression of ILP-2 (normalized to GAPDH) in the breast cancer serum samples compared with the non-cancer serum samples using Student’s *t*-test (*n* = 10, * *p* < 0.05).

Of the three proteins, only ILP-2 has been previously associated with cancer [16]; thus, we focused further studies on ILP-2. To validate the ILP-2 expression change in the breast cancer serum sample, we performed Western blot analysis on 10 breast cancer serum samples and 10 non-cancer serum samples (Figure 3A). Eight of the 10 breast cancer serum samples had increased ILP-2 levels compared to the non-cancer serum samples (Figure 3B). After normalizing the data to GAPDH levels, the average level of ILP-2 was 0.86 in the breast cancer serum samples and 0.19 in the non-cancer serum samples. The ILP-2 levels in the breast cancer serum samples were significantly higher than in the non-cancer serum samples using Student’s *t*-test (Figure 3C).

### 2.4. Comparison of 2DE with Five Types of Serum Samples

**Figure 4:**
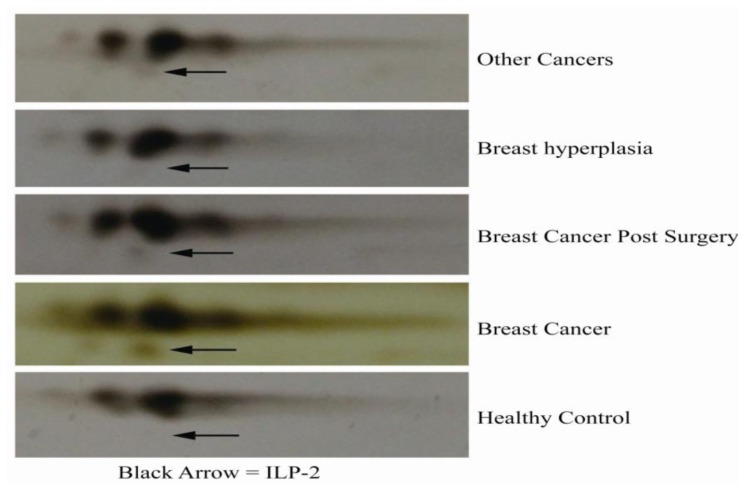
2D PAGE analysis of sera from five types of subjects. Serum samples are from subjects without cancer or with other types of cancers, galactophore hyperplasia, breast cancer post-surgery or breast cancer. The black arrow identifies ILP-2.

Serum samples from 35 subjects with breast cancer, other types of cancer, galactophore hyperplasia, breast cancer post-surgery or without cancer were analyzed with 2DE (Figure 4). ILP-2 was present in the serum samples from breast cancer, breast cancer post-surgery and other cancer subjects, whereas there was no ILP-2 present in the serum samples from healthy subjects or subjects with galactophore hyperplasia. Moreover, sera from subjects with breast cancer had increased ILP-2 levels compared to the other serum samples.

### 2.5. ELISA Analysis of ILP-2 Levels in Five Types of Serum Samples

**Figure 5:**
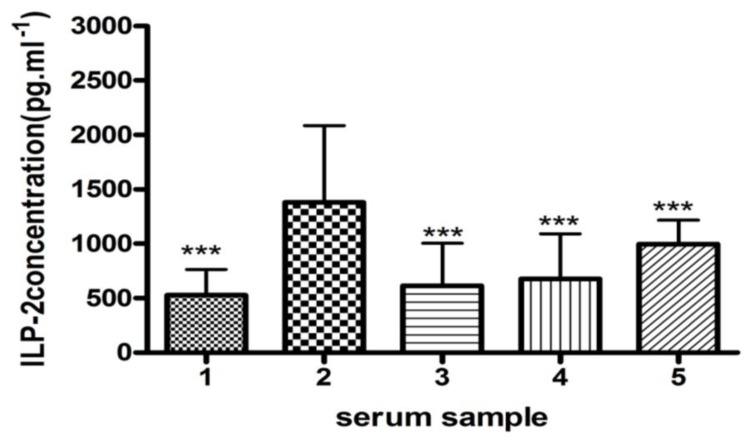
ILP-2 levels in the five types of sera measured by an ELISA. The numbers 1, 2, 3, 4 and 5 represent the serum samples from subjects with galactophore hyperplasia, breast cancer, breast cancer post-surgery, other cancer types and without cancer, respectively. An ANOVA was used to determine the statistical significance between the breast cancer subjects and the other samples (*n* = 35, ***p < 0.001).

An ELISA was performed to further validate the ILP-2 differences in the serum samples of 35 subjects (Figure 5). Although ILP-2 was present in all five types of serum samples, the sera from breast cancer subjects had significantly increased ILP-2 levels compared to the other serum samples. Furthermore, sera from post-surgery breast cancer patients had significantly less ILP-2 protein than the other serum samples, including the samples from breast cancer patients.

ILP-2 is the most recent apoptosis inhibitor to be identified [14,15] and has only been detected in the testis and in lymphoblastoid cells [14]. There are no previous studies on ILP-2 in breast cancer tissues and in sera from breast cancer patients.

From a medical perspective, a serum sample is the most informative proteome source, because numerous cells communicate through the secretion of soluble proteins into the blood [17,18]. Furthermore, serum analysis is a convenient method to diagnose breast cancer with a peripheral blood sample [19]. The correlation between ILP-2 and breast carcinogenesis and the potential of ILP-2 as a serological biomarker for breast cancer remain unclear.

We performed 2DE and MALDI-TOF analysis on 400 pooled samples of sera from breast cancer patients and 40 pooled samples of sera from healthy control subjects, and we identified ILP-2 expression in the breast cancer serum samples (Figures 1 and 2, Table 1). ILP-2 was present in the breast cancer serum samples, but not in the control serum samples.

Western blot analysis was performed on 10 breast cancer serum samples and 10 non-cancer serum samples to validate the correlation between ILP-2 and breast cancer (Figure 3). The results indicate increased levels of ILP-2 in the breast cancer serum samples compared to the non-cancer serum samples.

Then, we performed 2DE and MALDI-TOF analysis on 35 samples of sera from healthy subjects and subjects with other types of cancers, galactophore hyperplasia, breast cancer post-surgery and breast cancer according to previously described methods (Figures 2C and 4). The serum samples from subjects with breast cancer, breast cancer post-surgery and other cancer types contained ILP-2, whereas no ILP-2 was measured in the serum samples from galactophore hyperplasia and healthy control subjects. Moreover, the breast cancer serum samples had increased ILP-2 protein levels compared to the other serum samples.

Thereafter, we used an ELISA to analyze the 35 serum samples (Figure 5). The non-cancer serum samples were collected before the ELISA analysis. PMSF was added to the sample at 1 mM concentration, and the mixture was frozen at −80 °C. Despite the presence of ILP-2 in the serum samples from galactophore hyperplasia, breast cancer post-surgery, other types of cancer and non-cancer subjects, the ILP-2 protein levels were increased in the serum samples from breast cancer patients.

## 3. Materials and Methods

### 3.1. Ethical Statement

This study was approved by the Gannan Medical University Ethical Committee. Written informed consent was obtained from all patients before their participation in this study.

### 3.2. Patients

Breast cancer serum samples were collected from Stage 3 breast cancer patients who were diagnosed in accordance with the breast cancer pathology standard. The same procedure was applied to the serum samples from subjects with other types of cancers (Table 2). The average age of the breast cancer patients was 45 years, and the average age of the women with other types of cancers was 40 years.

Whole blood (5 mL) was collected from the cubital vein and then transferred to a clear, empty tube. The sample was incubated at room temperature for 2 h and then centrifuged at 1500× *g* for 10 min at 4 °C. The supernatant (80 μL) was transferred into an Eppendorf (EP) tube and 1 mM phenylmethylsulfonylfluoride (PMSF, Sigma, St. Louis, MO, USA) was added. The sample was stored in a freezer at −80 °C [20].

### 3.3. Serum Sample Preparation for Two-Dimensional Gel Electrophoresis (2DE)

The sera from 400 breast cancer patients and from 40 women without cancer were mixed separately. The mixtures comprised 50 μL serum from each sample. The mixed sera were centrifuged at 1500× *g* for 30 min at 4 °C. The supernatant was processed by using the Aurum™ Serum Protein Mini Kit (Bio-Rad, Hercules, CA, USA), according to the manufacturer’s instructions. The depleted sample (with albumin and IgG removed) was used for 2DE [21].

### 3.4. Protein Quantification

The protein concentration of the serum sample was quantified with the improved Bradford method [22].

### 3.5. 2DE

Samples of 35 μL of breast cancer sera and non-cancer sera (the undiluted sera) were transferred into separate, clear EP tubes. Then, 400 μL of the hydration solution (7 M urea, 2 M thiourea, 2% CHAPS (3-((3-cholamidopropyl)dimethylammonio)-1-propanesulphonate), 0.2% (*w*/*v*) Bio-Lyte (Bio-Rad, Hercules, CA, USA), 2% SB3-10 (*N*-decyl-*N*,*N*-dimethyl-3-ammonio-1-propanesulphonate) and 65 mM DTT (dithiothreitol, Sigma, St. Louis, MO, USA) was added to the sera and incubated at 25 °C for 2 h. The mixture was then added into a hydrate groove. An immobilized 17 cm pH gradient strip (pH 3–10, Bio-Rad, Hercules, CA, USA) was placed in the mixture and rehydrated at 25 °C for 16 h [23]. The isoelectric focusing (IEF) process involved the use of a 250 V linear ramp for 1 h, a 1000 V linear ramp for 1 h, a 4000 V linear ramp for 2 h, an 8000 V linear ramp for 3 h, an 8000 V rapid ramp for 10 h and a 500 V rapid ramp for 30 min with a PROTEIN IEF System (Bio-Rad, Hercules, CA, USA) [24]. After the IEF, each gel strip was equilibrated with 6 mL buffer I (6 M urea, 30% glycerol, 2% SDS, 0.375 M Tris-HCl pH 8.8 and 0.18 g/strip DTT) for 15 min and then with 6 mL buffer II (6 M urea, 30% glycerol, 2% SDS, 0.375 M Tris-HCl pH 8.8 and 0.225 g/strip iodoacetamide) for 15 min. The equilibrated gel strip was placed on top of a 12% sodium dodecyl sulphate polyacrylamide gel and then sealed with an agarose overlay (Bio-Rad, Hercules, CA, USA). The sodium dodecyl sulphate polyacrylamide gel electrophoresis (SDS-PAGE) was performed with 12 mA/per gel for 30 min and then with 25 mA/per gel until the bromophenol blue reached the bottom of the gels [25]. The gels were then stained with a silver-staining procedure [26,27].

The same method was applied to the 35 serum samples from the healthy control subjects and the subjects with other types of cancers, galactophore hyperplasia, breast cancer post-surgery and breast cancer.

### 3.6. Protein Spot Change Analysis and Peptide Mass Analysis

Before analyzing the different proteins in gels, the 2DE experiment was repeated twice under the same conditions to confirm the 2DE analysis reliability and reproducibility [16,28–30].

Changes in the protein spots of the 2DE maps between the breast cancer serum sample and the non-cancer serum sample were analyzed with the Melanie 3 Viewer. The spots that exhibited at least a two-fold change between the sample gels were excised and then transferred to a clear EP tube. The proteins were digested with the in-gel trypsin method, and the extracted peptides were dissolved in an equivalent volume of an α-cyano-4-hydroxycinnamic acid solution. After air-drying, the samples were placed on a stainless steel plate and desalted with 0.1% trifluoroacetic acid, and the peptides were identified with the Voyager DE Pro matrix-assisted laser desorption/ionization-time of flight (MALDI-TOF) mass spectrograph (Applied Biology System Company, Foster City, CA, USA) [31–33]. In the MALDI-TOF-mass spectrometry (MS) analysis, the extracted peptides were identified with the reflection mode and the positive ion spectrum. We applied adrenocorticotropic hormone for calibration and identified the peptide mass fingerprint using the Mascot Distiller (Matrix Science, London, UK). The detailed search parameters are listed in Table 3. The peptide match score was above 4, and the Mascot total score was above 64, which are regarded as statistically significant [34–36]. The same method was applied to the 35 serum samples from the healthy control subjects and the subjects with other cancer types, galactophore hyperplasia, breast cancer post-surgery and breast cancer.

### 3.7. Western Blot Analysis

A volume of 50 μL breast cancer serum or 50 μL non-cancer serum was separately diluted into 400 μL phosphate buffer. These sera mixtures were from 10 breast cancer patients and 10 healthy control subjects. The mixtures were centrifuged at 10,000× *g* for 20 min at 4 °C. The supernatant was collected, and then an equal volume of 2× loading buffer (100 mM Tris-HCl pH 6.8, 250 mM DTT, 10% β-ME, 4% SDS, 0.2% bromophenol blue and 20% glycerol) (Sigma, St. Louis, MO, USA) was added. The mixture was boiled for 10 min. A 20 μL/well sample was loaded onto 12% SDS-polyacrylamide gels [37]. The proteins were transferred to a polyvinylidene difluoride membrane [38]. Western blot analysis was performed by using a rabbit anti-human ILP-2 primary antibody (Abnova, Walnut, CA, USA) and a peroxidase-conjugated goat anti-rabbit IgG (Promega, Madison, WI, USA) [39]. An enhanced chemiluminiscence detection system (ECL-plus, Beytime, Haimen, China) was used to visualize the immunoreactive proteins via exposure to X-ray film (Tianguang, Tianjin, China). The rabbit anti-human ILP-2 (Promega, Madison, WI, USA) and rabbit anti-human GAPDH (Santa Cruz Biotechnology Inc., Santa Cruz, CA, USA) antibodies were diluted at 1:500 and 1:250, respectively. Signal intensity was quantified through densitometry. The signals were normalized to GAPDH [40–42].

### 3.8. Enzyme-Linked Immunosorbent Assay (ELISA) Analysis

A total of 35 serum samples from healthy subjects and subjects with other cancer types, galactophore hyperplasia, breast cancer post-surgery and breast cancer were diluted 40,000-fold and then analyzed according to the kit instructions (USCNLIFE, Missouri, TX, USA) [43,44]. The ELISA was performed on every serum sample in triplicate. The optical density of each sample was measured with a microplate reader at 450 nm [45,46].

### 3.9. Statistics Analysis

The results were presented as *X* ± SD. Statistical analysis was performed using Student’s *t*-test, analysis of variance (ANOVA) or Tukey’s test with SPSS 11.5. A value of *p* < 0.05 was considered statistically significant.

## 4. Conclusions

In this study, we analyzed the ILP-2 levels of serum samples from women without cancer and women with breast cancer, galactophore hyperplasia, breast cancer post-surgery and other types of cancer. Although a significant amount of work needs to be conducted concerning ILP-2, these results suggest that ILP-2 is a novel serum biomarker for breast cancer. Our work states that ILP-2 can be a breast cancer biomarker, but can also be detected in other cancer samples. Therefore, related validation work needs to continue in further analyses.

## Figures and Tables

**Table 1:** Proteins identified in the breast cancer serum sample.

No.	Protein	Matched peptides	GenBank accession no.	Top score	Significance	Function
1	Fxia Catalytic Domain	5	gi|56967288	64	*p* < 0.05	The Fxia catalytic domain in complex with ecotinm84r
2	HP protein	5	gi|34785974	74	*p* < 0.05	Haptoglobin alpha (2FS)-beta precursor
3	ILP-2	9	gi|15042064	54	*p* < 0.05	Testis-specific inhibitor of apoptosis

**Table 2:** Serum sample data.

Sample source	Type of sample	Number of samples	Type of analysis
The Third Hospital of Kunming Medical College, Kunming, China	Breast cancer	400	2DE, MALDI TOF MS
	Non-cancer	40	2DE, MALDI TOF MS

The First Hospital of Gannan Medical College, Ganzhou, China	Breast cancer	10	WB
		35	2DE, ELISA

	Non-cancer	10	WB
		35	2DE, ELISA

	Galactophore hyperplasia	35	2DE, ELISA

	Breast cancer post-surgery	35	2DE, ELISA

	Other cancer types	35	2DE, ELISA

**Table 3:** Search parameters.

Parameter types	Search parameters
Type of search	Peptide Mass Fingerprint
Enzyme	Trypsin
Variable modifications	Carbamidomethyl (C)
Mass values	Monoisotopic
Protein Mass	Unrestricted
Peptide Mass Tolerance	±0.15 Da
Peptide Charge State	1+
Max Missed Cleavages	2
Number of queries	13
